# Transseptal Puncture Guided by Three-Dimensional Electroanatomical Mapping: Early Experience Using a Simplified Approach in Adults with Congenital Heart Disease

**DOI:** 10.3390/jcm12134491

**Published:** 2023-07-05

**Authors:** Fu Guan, Matthias Gass, Florian Berger, Deniz Akdis, Firat Duru, Thomas Wolber

**Affiliations:** 1 Department of Cardiology, Arrhythmia and Electrophysiology Division, University Heart Center Zurich, 8091 Zurich, Switzerland; fu.guan@usz.ch (F.G.);; 2 Children’s Research Center, Zurich University Children’s Hospital, 8032 Zurich, Switzerland; 3 Center for Integrative Human Physiology, University of Zurich, 8091 Zurich, Switzerland

**Keywords:** transseptal puncture, fluoroscopy, congenital heart disease, catheter ablation, electroanatomical mapping

## Abstract

Aims: The widespread use of three-dimensional (3D) mapping systems and echocardiography in the field of cardiac electrophysiology has made it possible to perform transseptal punctures (TSP) with low or no fluoroscopy. However, such attempts in adults with congenital heart disease (ACHD) who have previously undergone surgical or interventional treatment are limited. Therefore, we sought to explore the feasibility and safety of an approach to perform zero- or low-fluoroscopy TSP in ACHD patients undergoing left atrial cardiac ablation procedures. Methods and results: This study included 45 ACHD patients who underwent TSP for ablation of left-sided tachycardias (left atrium or pulmonary venous atrium). Computed tomography (CT) of the heart was performed in all patients prior to ablation. 3D mapping of the right-sided heart chambers before TSP was used to superimpose the registered anatomy, which was subsequently used for the mapping-guided TSP technique. TSP was performed with zero-fluoroscopy in 27 patients, and the remaining 18 patients had a mean fluoroscopy exposure of 315.88 ± 598.43 μGy.m^2^ and a mean fluoroscopy duration of 1.9 ± 5.4 min. No patient in this cohort experienced TSP-related complications. Conclusion: Our study describes a fluoroscopy-free or low-dose fluoroscopy approach for TSP in ACHD patients undergoing catheter ablation of left-sided tachyarrhythmias who had been previously treated surgically or interventionally due to congenital heart defects. By superimposing 3D electroanatomic mapping with cardiac CT anatomy, this protocol proved to be highly effective, feasible and safe.

## 1. Introduction

The introduction of three-dimensional (3D) electroanatomic mapping (EAM) systems and the use of intracardiac echocardiography have made it possible to perform a transseptal puncture (TSP) for cardiac ablation of left-sided arrhythmias with very low or no fluoroscopy [1,2]. Despite this, the number of patients with adult congenital heart disease (ACHD) requiring catheter ablation is constantly increasing, yet clinical data on fluoroscopy-free TSP in this population are limited. Arrhythmias in ACHD patients may be the result of fibrosis related to the underlying disease, hemodynamic changes or associated with surgical correction or interventional prosthetic repair procedures, as well as aging-related arrhythmias, such as atrial fibrillation [3,4]. TSP for catheter ablation of left-sided arrhythmias in ACHD patients is currently performed mainly under fluoroscopy guidance and transesophageal or intracardiac echocardiography [5,6]. Therefore, we sought to explore a simplified and safe fluoroscopy-free approach for TSP in ACHD patients, using 3D EAM combined with computerized tomography (CT) scanning-based reconstruction of cardiac anatomy.

## 2. Methods

### 2.1. Patient Population

Forty-five ACHD patients after surgical correction or interventional repair, who were scheduled to undergo arrhythmia ablation in the left or pulmonary venous atrium at the University Heart Center Zurich, were included in this study. Prior to ablation, CT scanning of the heart was performed in all patients. Arrhythmias included left-sided atrial tachycardias and atrial flutters, atrioventricular (AV) reentry tachycardias due to accessory pathways, and atrial fibrillation. Patients with preexisting interatrial connections, such as persistent patent foramen ovale (PFO) or baffle leak, were excluded. Patients with cardiac implantable electronic devices (CIEDs) were also excluded. Upon giving consent, patients were informed of the benefits, risks and alternative approaches. The duration of the procedure was documented on a step-by-step basis, from the initial venous access to the final removal of all sheaths. During the procedure, TSP was performed, employing a “non-radiation where possible, low radiation where necessary” approach. The use of fluoroscopy was at the discretion of the operator to ensure maximum patient safety. The feasibility and safety of TSP, including intra- and periprocedural complications, were assessed.

### 2.2. 3D Mapping-Guided TSP Protocol

The procedure was generally performed under conscious sedation. General anesthesia was used in patients undergoing atrial fibrillation ablation. Ultrasound-guided femoral venous or arterial access was performed in all patients. CARTO 3D EAM system (Biosense Webster, Irvine, CA, USA) TSP was performed in combination with the Agilis long sheath with a dilator (Abbott, Chicago, IL, USA), the BRK™ transseptal needle (Abbott, IL, USA), and the SafeSept guidewire (Pressure Products, San Pedro, CA, USA). In the presence of a postsurgical prosthetic septum, such as a Fontan conduit or fabric septal patch/occluder, an NRG transseptal needle (Boston Scientific, St. Paul, MN, USA) was used to facilitate TSP.

**Figure 1:**
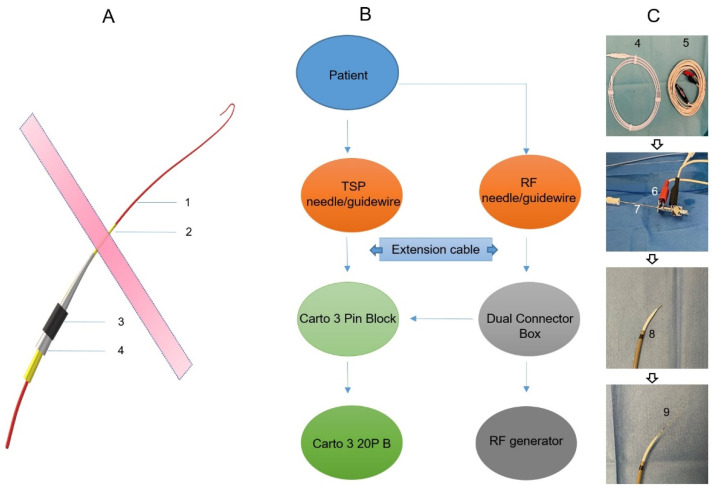
A schematic of the equipment setup for visualizing the transseptal needle/guidewire. (**A**): A schematic of the transseptal delivery system. 1: Guidewire; 2: Transseptal needle; 3: Steerable long sheath; 4: Dilator. (**B**): A schematic of the equipment setup for visualizing the transseptal needle (A2)/guidewire (A1). (**C**): Photos of the related materials for a 3D visualized transseptal puncture. 4: Guidewire; 5: Extension cable; 6: Stackable pin jumper connecting transseptal needle (7); 8: Steerable long sheath with a dilator and transseptal needle inside; 9: Steerable long sheath with a dilator, transseptal needle, and guidewire inside.

Prior to the procedure, the CARTO system and the TSP system were set up for mutual communication to enable 3D visualization. RF pins, stackable 2 mm pin jumpers, and quadrupole catheters were attached to the CARTO pin block, as shown in the schematic in Figure 1. A preset quadruple catheter definition (20B 4F quad 2-5-2 mm fixed) was selected in CARTO software Version 7 to allow for the visualization of the TSP needle and guidewire. Subsequently, the electrode tip of the TSP needle/guide wire was visualized on the CARTO mapping system as a feature of the primary electrode. The anatomical reconstruction was performed using the CT scan and the CartoMerge module. Reconstructed 3D cardiac anatomy included atria and ventricles, ascending aorta, pulmonary artery, and coronary sinus (CS).

During the procedure, right-sided intracardiac mapping was performed with a 20-polar Pentaray mapping catheter (Biosense Webster, CA, USA). A decapolar catheter (Biosense Webster, CA, USA) was introduced into the CS position. The position of the His bundle as an anatomical reference was also labeled at multiple points by the mapping catheter. Special consideration was given to mapping the interatrial septal portion of the heart for TSP.

**Figure 2:**
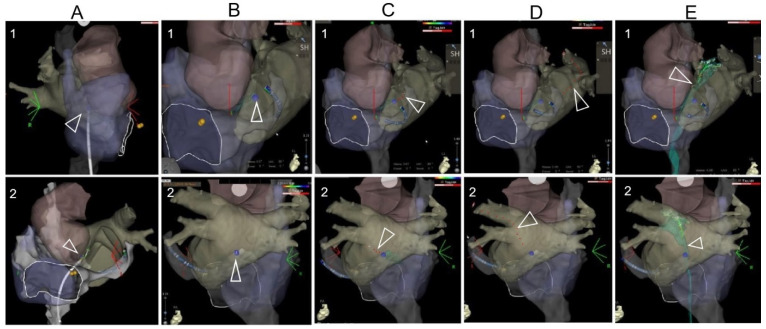
The TSP images under the visualization of a 3D mapping system. (**A**): After the 3D mapping of the right-sided anatomy, the CT anatomy was superimposed. The RF catheter was tracked during its moving from the superior part of the mapping anatomy. (**B**): The location of the TSP was identified and annotated on the 3D mapping anatomy. (**C**): The TSP needle was visualized and positioned on the annotated TSP point through the steerable sheath. (**D**): After visualization of needle puncture through the septum, it was replaced by the guidewire. The guidewire was tracked during its advancement to the PV on the 3D reconstructed anatomy. Note that 1 and 2 are modified projections for better visualization of the superimposed reconstructed anatomy (usually modified RAO and modified LL or PA projections). (**E**): After the TSP, the mapping catheter was introduced to the left chamber via the long sheath, and a FAM (fast anatomical mapping) of TSP was performed, shown as green color.

The 3D mapped right-sided cardiac anatomy was registered and superimposed with the CT scan reconstructed 3D anatomy in the CARTO system, which permitted real-time visualization of the TSP needle tip and guiding wire within this comprehensive 3D model. This visualization was automatically updated to alter 3D map projections on the CARTO system. Amid this maneuver, the aortic root, the pulmonary veins, and the interatrial septum were used as landmarks, which were repeatedly visualized with a slight clockwise or counterclockwise rotation of the CT scan-merged 3D images. Prior to performing TSP, the TSP location was annotated on the mapping system with the ablation catheter to avoid adjacent tissue injury. Puncture with the TSP needle into the left/pulmonary venous atrium and the following advancement of the guiding wire into the left/right superior pulmonary vein was visualized by the preset 3D mapping system, shown in Figure 2 and Supplemented Video. Bedside transthoracic echocardiography was used to exclude pericardial effusion prior to and after the procedure. All patients were followed up for adverse events perioperatively and postoperatively until 4 weeks after the ablation procedure. Pericardial effusion requiring intervention was defined as cardiac tamponade.

### 2.3. Ethical Approval

This study complied with the Declaration of Helsinki and was approved by the local Ethics Committee (Cantonal Ethics Committee Zurich, Nr. 2016-00116). All patients gave informed written consent for the procedure and the use of clinical data for scientific study.

### 2.4. Statistical Analysis

Baseline characteristics are reported as means ± standard deviations (SD), and categorical characteristics as counts and percentages. Parameters associated with TSP are reported as numbers and percentages. For the likelihood ratio test, the Chi-Square test was used and a *p*-value < 0.05 was considered significant. All analysis was performed using SAS Statistical software (version 9.4; SAS Institute Inc., Cary, NC, USA).

## 3. Results

### 3.1. Patient Characteristics

The underlying congenital heart disease of the 45 ACHD patients (49 ± 16 years of age, 71% male) included atrial septal defect (ASD), atrioventricular septal defect (AVSD), dextro-transposition of great arteries (d-TGA), congenitally corrected transposition of great arteries (ccTGA) with tricuspid atresia (TA), and double inlet left ventricle (DILV) with TA. Fabric (e.g., Gore-Tex (W.L. Gore, Flagstaff, AZ, USA), Dacron (Bard^®^ Savage^®^ filamentous knitted polyester fabric, Bard Peripheral Vascular Inc., Tempe, AZ, USA)) patches and occluders (e.g., Core Cardioform (W.L. Gore & Associates Inc., Newark, DE, USA), Amplatzer (St. Jude Medical, Minneapolis, MN, USA)) (76%) or pericardial patches (24%), as a partial or full substrate of the septum, were present in all patients due to a surgically corrected/palliated condition or an interventional repair a mean (± SD) of 25 ± 16 years before (Table 1).

### 3.2. 3D Mapping Guided TSP Parameters

The average time needed for CartoMerge and superimposition of the 3D mapping with CT scan reconstructed anatomy was 7 ± 5 min. Single 3D-guided TSP was successful in all 45 cases, among which 27 procedures were performed without fluoroscopy. No major complications were observed in any patient (Table 2).

**Figure 3:**
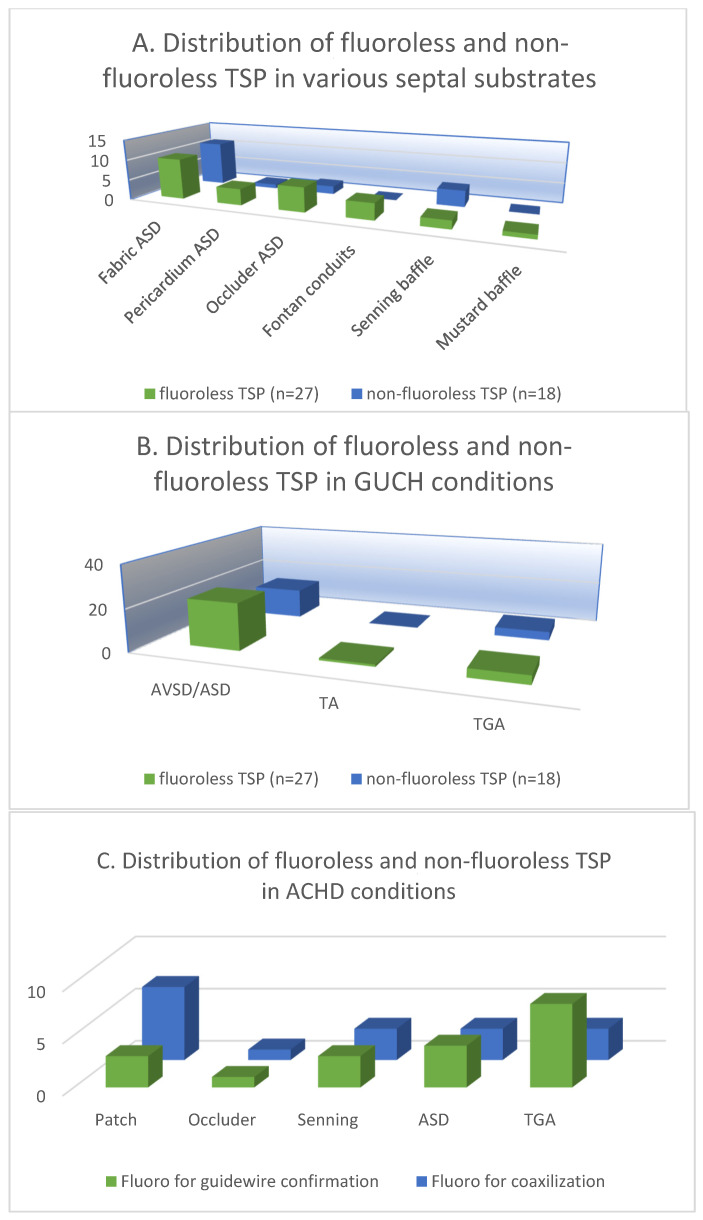
The distribution of fluoroless and non-fluoroless TSP in various septal substrates (**A**) and ACHD conditions (**B**). (**C**) The distribution of fluoroscopy-guided guidewire confirmation and coaxilization in patients with various septal substrates and ACHD conditions.

The patients were categorized and analyzed according to the atrial septal substrate in the 27 cases of fluoroless TSP and 18 cases of non-fluoroless TSP. The 3D mapping-guided TSP was performed in a total of 27 (60%) fabric septa in both groups (35% vs. 24%). The fabric septa included ASD/AVSD septal occluders, extracardiac Fontan conduits and baffles after the Mustard operation. TSP was performed in a total of 11 (24%) pericardial/atrial septa in patients with surgically repaired ASD/AVSD and Senning operation (13% vs. 11%). Furthermore, the TSP parameters were analyzed by the congenital conditions of the patient, among which there were 36 (80%) cases of AVSD/ASD (49% vs. 31%), 1 (2%) case of TA (2% vs. 0%), and 8 (18%) cases of TGA (9% vs. 9%) (Figure 3A,B).

In the 18 patients who underwent TSP with fluoroscopy, the mean fluoroscopy time for TSP was 1.9 ± 5.4 min and the dose area product was 315.88 ± 598.43 μGy.m^2^. Multivariate analysis showed that the application of fluoroscopy was not associated with the clinical characteristics of the patients as well as the status of ACHD and the IAS substrates (Table 3). Despite our relatively small sample size, we reviewed the fluoroscopic data and divided the application of fluoroscopy during TSP into two groups: for guidewire advancement into PVs and long sheath-needle-wire coaxialization. Among the 18 patients, both applications were applied in 3 patients with fabric patch repaired ASD, 3 TGA patients with post-Senning IAS, and 1 patient with ASD occluder (Amplatzer) (Figure 3C). Moreover, no significant differences were found between patients who underwent TSP without fluoroscopy and those who had fluoroscopy in terms of baseline clinical characteristics, IAS substrate, ACHD condition, years of surgical or interventional treatment, the presence of previous TSP, or types of TSP needles (*p* > 0.05).

## 4. Discussion

### 4.1. Main Findings

This study demonstrates that a 3D mapping-guided TSP approach is feasible and safe in ACHD patients after surgical or interventional treatment with no or low-dose fluoroscopy. This can be performed by real-time visualization of the TSP needle and guidewire within the 3D electroanatomic map, superimposed with a pre-procedurally acquired cardiac CT anatomy. The target location for a successful TSP can be defined after taking into account all adjacent anatomical structures to reduce possible complications.

A transseptal puncture is usually performed under the guidance of conventional fluoroscopy or TEE. Previous studies have combined CT-scan reconstructed cardiac anatomy with fluoroscopic acquisition with Carto Univu, which allows for the overlay of catheter-acquired cardiac maps into fluoroscopy, which can then be used as a reference for cardiac anatomy during atrial fibrillation ablation [7]. Given the increasing awareness of radiation exposure in interventional electrophysiology, several approaches for fluoroscopy-free procedures have been proposed [8,9,10]. Recently, fluoroless TSP has been attempted in some centers and demonstrated as a feasible approach [1,2]. In these studies, the fossa ovalis (FO) of the atrial septum was used as the main landmark for the localization of the puncture site. The local anatomy of the atrial septal FO could be precisely depicted during right atrial 3D mapping. During the procedure, the mapping catheter was slowly pulled back from the superior vena cava of the 3D mapping image, until achieving the same local tenting image as in fluoroscopy-guided two-dimensional TSP. These approaches were successfully completed in combination with TEE guidance or local electrical potential identification.

Based on the real-time visualization of the tip of the catheter in the target cardiac chamber, an accurate 3D reconstruction of the anatomy derived from a CT scan or cardiac magnetic resonance imaging (CartoMerge) can be superimposed, with the 3D mapping anatomy, and used during the procedure. Thus, 3D EAM systems have not only greatly improved the efficacy and success of cardiac mapping and catheter ablation, but have also been further applied to the field of cardiac pacing and other cardiac interventions such as endomyocardial biopsy (EMB) [11,12,13]. According to these investigations, the pacing electrodes or biopsy forceps connected to the mapping system can be systematically identified and displayed within the 3D maps. Promising results of 3D mapping-guided pacemaker electrode implantation have been reported with safety as well as fluoroscopy reduction [11,14,15]. Similarly, EMBs guided by 3D mapping of the target chamber were proven to be safe [13]. These studies demonstrated that the connection and identification of external components such as wires, needles, and forceps to the mapping system are feasible and safe.

It is important to note that a large percentage of ACHD patients have abnormal interatrial septa with altered tissue substrate due to surgical or interventional repair or as a result of hemodynamic and structural changes associated with the underlying congenital condition [16,17,18,19]. In such patients the optimal TSP location at the FO may be difficult to detect and during the conventional “pull back” of the long sheath with the TSP needle inside, the “protrusion technique” cannot always be applied [1]. Recent studies have demonstrated the feasibility and safety of a successful TSP in patients with previous ASD occluder or surgically repaired interatrial septum. A selected puncture site, such as the posteroinferior part of the septum or direct puncture of the ASD occluder guided by fluoroscopy combined with/without intracardiac ultrasound, has been shown to be effective [20]. Moreover, in patients with d-TGA after Mustard or Senning procedures or single ventricle after Fontan surgery, TSP can be successfully performed through conduits, pericardial patches, or synthetic patches with low risk for major complications [21]. In addition, the use of a radiofrequency (RF) needle in hard-to-penetrate septal substrates and SafeSept™ percutaneous guidewires during TSP have been proven safe and effective for obtaining left atrial access for electrophysiologic procedures in patients with complex ACHD [22,23]. However, it is worth noting that all these procedures still require an accountable amount of fluoroscopy exposure. Ultimately, comprehensive management of the ACHD population is a lifelong task. Patients are exposed to increasing amounts of low-dose ionizing radiation (LDIR) in the clinic, not only during pre-surgical evaluation and post-operative follow-up imaging but also related to various transcatheter procedures. This raises concerns about the associated risk of malignancy in this young adult population. Therefore, any measure to reduce further radiation exposure is beneficial in preventing cancer for patients as well as for interventional electrophysiologists. Cardiac electrophysiologists are responsible for ensuring that radiation exposure is reduced maximally during EP procedures without sacrificing the quality of patient care [24].

In our present approach, the 3D map of the right-sided heart was superimposed with a CT 3D reconstruction of the cardiac anatomy integrated into the CARTO system. This allowed visualization of the important anatomical landmarks, such as the aortic root and the relationship between the posterior atrial wall and the atrial septum, which are difficult to see in real-time from multiple projections under conventional fluoroscopy and TEE. With the present CARTO-guided TSP approach, we are able to accurately determine the optimal position of the TSP and its relationship to the adjacent structures. By linking the CARTO system to the TSP set, the TSP needle and guidewire could be visualized within the cardiac anatomical structures in real-time. Due to the overall diversity and complexity of the congenital conditions of the patients, it was not possible to completely avoid fluoroscopy, although the lack of or low exposure to fluoroscopy makes this approach attractive. The passage of the atrial septum was difficult to achieve in some cases, either due to tissue changes with increased resistance after surgical or interventional repair of the septum, or due to anatomical conditions after the atrial switch or Fontan operations. Moreover, in some cases, it was impossible to smoothly advance the guidewire into LA/SVA or the target PVs. In these cases, by confirming the coaxiality of the puncture system or excluding the guidewire advancing to the pericardial cavity, using fluoroscopy or even a small amount of contrast injection was adopted. Therefore, the operator applied fluoroscopy in some cases in order not to compromise patient safety. Our present analysis of the cohort did not reveal any association between fluoroscopy application and patients’ clinical risk factors such as age, BMI, comorbidities, etc., as well as ACHD status, which includes years of surgical or interventional treatment, IAS substrates, and altered cardiac structures. Detailed correlation analysis requires further prospective studies with large samples.

### 4.2. Clinical Implications

In our study, reliable real-time tracking of the TSP needle position and guidewire movement in a 3D mapping system could be achieved during catheter ablations in ACHD patients. In this regard, it may be hypothesized that a 3D-guided fluoroscopy-free TSP approach may be feasible in patients with normal cardiac anatomy whose fossa ovalis can be clearly mapped and is therefore suitable as a target for TSP. Moreover, in patients with severe heart rotation and malformation due to systemic disease, such as chronic obstructive pulmonary disease, diaphragmatic elevation or sternal deviation, for whom TSP may be complicated even with the guidance of fluoroscopy and/or TEE, our TSP protocol might possibly be of help.

### 4.3. Study Limitations

This is a single-center study with a relatively small sample size. The included patients with ACHD had different congenital diseases and surgical histories, so the TSP technique and anatomy could not be standardized, making it impossible to draw a uniform atlas related to cardiac anatomy.

## 5. Conclusions

In conclusion, in ACHD patients with surgically or interventionally altered interatrial septum, 3D EAM combined with 3D reconstructed anatomy from cardiac CT scans can safely guide TSP. This 3D EAM-guided TSP procedure may be performed with zero or very low fluoroscopy exposure.

## Figures and Tables

**Table 1:** Clinical characteristics of the patients.

	Total TSP	Zero Fluoroscopy	Low Fluoroscopy
Patients number	45	27	18
Average age (years)	49 ± 16	51 ± 15	47 ± 13
Male sex (n %)	32 (71)	20 (22)	12 (67)
Average body mass index (kg/m^2^)	27.6 ± 5.0	28.3 ± 5.0	26.5 ± 8.0
Arterial hypertension (n %)	9 (20)	6 (22)	3 (17)
Pulmonary hypertension (n %)	14 (31)	9 (33)	5 (28)
Average LVEF (%)			
≥55%	28 (62)	20 (74)	8 (44)
45–55%	14 (31)	5 (19)	9 (50)
<45%	3 (7)	2 (7)	1 (6)
Arrhythmias (%)			
Atrial fibrillation *	17 (38)	11 (41)	6 (33)
Atrial flutter *	3 (6)	2 (7)	1 (6)
Atrial tachycardia(IART)	23 (51)	12 (44)	11 (61)
WPW	2 (4)	2 (7)	0 (0)
Congenital heart disease (%)			
ASD	20 (44)	11 (41)	9 (50)
AVSD	18 (40)	13 (48)	5 (28)
TA	2 (4)	2 (7)	0 (0)
d-TGA	8 (18)	4 (4)	4 (22)
ccTGA	2 (4)	2 (7)	0 (0)
Oral anticoagulants			
NOAC (%)	22 (49)	17 (63)	5 (28)
VKA (%)	21 (47)	8 (30)	13 (72)
Aspirin (%)	2 (4)	2 (7)	0 (0)

* Patient with AT due to previous PVI was grouped as atrial fibrillation. Atrial flutter was CTI-dependent flutter for d-TGA in this ACHD population.

**Table 2:** Anatomical and TSP procedural parameters.

	Total (*n* = 45)	Zero Fluoroscopy (*n* = 27)	Low Fluoroscopy (*n* = 18)
Atrial septal parameters			
Pericardium IAS (%)	5 (11)	4 (15)	1 (6)
Occluder (%)	8 (18)	6 (22)	2 (11)
Gore Cardioform	1 (2)	1 (4)	0 (0)
Amplatzer	7 (16)	5 (19)	2 (11)
Synthetic IAS (%)	21 (47)	10 (37)	11 (61)
Baffle IAS (%)			
Senning (%)	6 (13)	2 (7)	4 (22)
Mustard (%)	1 (2)	1 (4)	0 (0)
Modified Fontan (%)	4 (9)	4 (15)	0 (0)
Previous TSP (%)	12 (27)	8 (30)	4 (22)
TSP procedural parameters			
TSP number	45	27	18
TSP needle	45	27	18
Normal needle (%)	36 (80)	22 (81)	14 (78)
RF needle (%)	9 (20)	4 (15)	5 (28)
TSP time (min) *	25 ± 7	25 ± 7	26 ± 8
TSP related complications	0	0	0

* TSP time was recorded from the beginning of right-sided 3D mapping to the end of TSP after the mapping catheter was introduced to the left chamber via a long sheath.

**Table 3:** The odds ratio for fluoroscopy application during TSP.

Variables	OR	95% CI		*p*
Age (years)	1.013	0.977	1.051	0.478
BMI (kg/m^2^)	1.087	0.949	1.245	0.2293
Hypertension	2.105	0.475	9.338	0.3273
Previous TSP	1.474	0.369	5.885	0.5831
LVEF (%)	1.225	0.3	4.995	0.7772
ACHD conditions	0.602	0.166	2.181	0.44
IAS substrates	1.123	0.76	1.66	0.5605
Years of surgical/interventional treatment	1.01	0.972	1.048	0.6205
TSP needle	0.452	0.103	1.987	0.2933

*p* < 0.05 is considered as statistically significant. CI, Confidence interval.

## Data Availability

The data that support the findings of this study are available from the corresponding author, upon reasonable request.

## Supplementary Materials

The following supporting information can be downloaded at: https://www.mdpi.com/article/10.3390/jcm12134491/s1, Supplemented Video.
